# New-onset diabetes in COVID-19: The molecular pathogenesis

**DOI:** 10.37796/2211-8039.1389

**Published:** 2023-03-01

**Authors:** Desak Made Wihandani, Made Lady Adelaida Purwanta, W. Riski Widya Mulyani, I Wayan Ardyan Sudharta Putra, I Gede Putu Supadmanaba

**Affiliations:** Department of Biochemistry, Faculty of Medicine Udayana University, Sanglah General Hospital, Denpasar, Bali, Indonesia

**Keywords:** COVID-19, SARS coronavirus, Diabetes, ACE2

## Abstract

Diabetes mellitus (DM) is still a challenging metabolic disease worldwide. In the current situation, the world is facing a COVID-19 pandemic due to SARS-CoV-2 infection. DM is one of the comorbid conditions that can worsen the severity of the COVID-19 condition. Surprisingly, SARS-CoV-2 infection can induce new-onset diabetes, a condition in which acute hyperglycemia occurs and may develop into a complication in nondiabetic patients. Angiotensinconverting enzyme 2 (ACE2) is a crucial entry factor for SARS-CoV-2 infection. ACE2 will bind to the spike protein of SARS-CoV-2, potentially initiating a damaging process in many tissues in the human body, including metabolic tissues. This mechanism suggests a potential role of ACE2 in the pathogenesis of diabetes since ACE2 has been proven to localize in essential metabolic tissues, one of which is the acini and islets part of the pancreas. This interrelated ACE2 in COVID-19 and DM is thought of as the mechanism that induces new-onset diabetes in COVID-19 patients. This review will thoroughly describe the current findings and theories regarding the molecular mechanism of SARS-CoV-2-induced new-onset diabetes and the possible therapeutic intervention.

## 1. Background

Diabetes Mellitus (DM) is still a major metabolic problem and the leading cause of morbidity and mortality worldwide. In 2014, World Health Organization (WHO) reported that 422 million adults had diabetes and its prevalence increases yearly [1]. In 2020, we were also challenged by the COVID-19 pandemic. COVID-19 is a disease due to the infection of Severe Acute Respiratory Syndrome-Coronavirus-2 (SARS-CoV-2) [2]. WHO reported that 83 million with COVID-19 confirmed positive cases and about 1.8 million confirmed death [3]. COVID-19 symptoms vary from mild to severe; about 90% of patients showed more than one symptom, and the three most prevalent symptoms are fever, fatigue, and cough [4]. Diabetes has been identified as a risk factor for many infection cases [5,6]; thus, it is suggested as a comorbidity that increases the severity of the COVID-19 infection [7–10].

Uniquely, some cases have been reported with newly diagnosed DM in COVID-19 positive cases without any history of DM [11,12]. Alsadhan et al. reported five patients admitted to the hospital with diabetic ketoacidosis (DKA) and positive result on real-time reverse transcription-polymerase (RT-PCR) COVID-19. Three of them had a DM history, and the others were diagnosed with new DM after being admitted to the hospital with a high level of HbA1c [11]. Another case report reported three patients, one diagnosed with DKA and positive for COVID-19. Interestingly, the other two patients showed classic DM symptoms like polydipsia and polyuria post-infected with COVID-19 [12]. In this condition, COVID-19 infection is aggravated by the acute hyperglycemia onset, which, if not treated properly, could potentially lead to fatal complications in the patient without a history of diabetes. With such unpredictable and rapid disease progression, this phenomenon has become quite a unique and urgent concern that needs to be unveiled. This review will discuss the new onset of DM in COVID-19 infection more profoundly and show how COVID-19 and DM interact with each other in the molecular aspect.

## 2. The role of ACE2 receptors in SARS-CoV-2 infection

Angiotensin-converting enzyme 2 (ACE2) has long been a key receptor for the SARS coronavirus. Becoming the first homolog of ACE with a homology sequence of 42%, ACE2 was first found in human heart failure [13]. SARS-CoV and SARS-CoV-2 utilize ACE2 as an entry receptor by binding it with surface protein S and may partly explain the pathogenesis and predilection of COVID-19 [14–18]. The identification of ACE2 as an entry receptor for SARS-CoV-2 was primarily facilitated by its similar role in SARS-CoV, which was revealed in 2003. Using the fusion protein technique, Li et al. unveiled that SARS-CoV efficiently bound ACE2 through S1 protein, and the soluble ACE2 blocked S1 domain-ACE2 interaction [19–22]. However, soluble ACE1 did not produce a similar result [21]. It also revealed that the anti-ACE2 antibody blocked SARS-CoV replication in Vero E6 cells from African green monkeys, but antiACE1 had no effect [21]. These results provided a strong foundation for identifying the SARS-CoV-2 receptor.

**Fig. 1:**
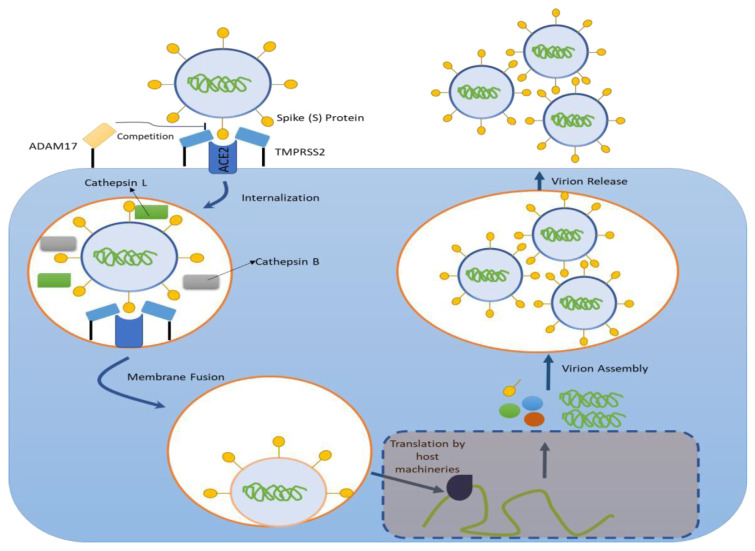
The infection process of host cells by SARS-CoV-2. Similar to SARS-CoV, SARS-CoV-2 uses ACE2 as its receptor and viral internalization begins with the interaction between spike (S) protein and ACE2, primed by TMPRSS2. After internalization, endosomal proteases facilitate the fusion between viral membrane and endosomal membrane. The viral RNAs use host machinery to translate their genetic information into functional viral proteins which then assemble themselves into a new endosome. The exocytosis process finally releases the new virions to the extracellular space.

Accordingly, ACE2 also became the primary receptor of SARS-CoV-2, which was revealed through extensive in vivo studies [23–27]. Transgenic mice with ACE2 deficiency had much less viral load and viral replication than the control mice [23,25]. These mice also experienced milder pulmonary alterations compared to the wild-type mice. Additionally, mice with human ACE2 overexpression also developed a higher rate of severe symptoms, which resemble human patients. Interestingly, the symptoms worsened when the mice were only injected with SARS-CoV-2 spike protein [27]. Consistently, the administration of recombinant soluble ACE2 effectively blocked the interaction between spike (S) protein and ACE2, highlighting its therapeutic potential for both SARS and COVID-19 [26]. Together, all of this evidence suggests the pivotal role of ACE2 in SARS-CoV-2 infection and, possibly, pathogenesis (see Fig. 1, illustration adapted from Pang et al. [26]).

### 2.1. Spike (S) protein and cellular proteases mediate SARS-CoV-2 entry

The spike (S) proteins of SARS-CoV and SARS-CoV-2 are similar to 76.5% similarity in amino acid sequences [28]. SARS-CoV-2's S protein has 1273 amino acids with two crucial domains, referred to as S1 and S2. Other important parts of the S protein are the 19 AA Nterminal, which serves as a signal peptide, and the C-terminal's short cytoplasmic and short transmembrane domains. The S1 can be divided into an N-terminal domain (NTD) and a Cterminal domain (CTD); both serve as receptor-binding domains. In both SARS-CoV and SARS-CoV-2, these domains recognize ACE2, which serves as the virus's entry receptor. Despite the difference between the S protein of both viruses, their 3D structure is similar, which underlies the function and receptor's similarity [14,15,29].

In order to facilitate its entry, the S protein is primed by cellular proteases such as endosomal cysteine proteases (cathepsin B and L) and transmembrane serine protease 2 (TMPRSS2) [15,30–33]. This process is very similar to the cellular entry of SARS-CoV. Proteolysis is essential for SARS-CoV-2 entry, and both S1 and S1 proteins need to be cleaved to initiate the viral entry process [34]. Interestingly, the cleavage site of SARS-CoV-2 is slightly different from SARS-CoV with a new, conserved insertion sequence between S1 and S2, which is recognized by Furin, a kexin-like subfamily of proprotein convertases [35]. Another difference is the arginine residues found in the S1/S2 cleavage site, but the importance of these differences needs to be investigated [36].

The importance of endosomal cysteine proteases is reported in several studies that showed that modification of endosomal pH inhibited SARS-CoV-2 entry, which is likely due to the inactivation of endosomal proteases [15]. On the other hand, camostat mesylate, a TMPRSS2 inhibitor, only partly inhibited SARS-CoV-2-S cellular entry. Finally, the viral entry is entirely blocked if TMPRSS2 and endosomal cysteine proteases are inhibited [15].

TMPRSS2 is important for viral fusion protein activation during cellular entry, specifically by cleaving and activating subunit S1, facilitating viral attachment to the target cell plasma membrane [15,30,31]. Pathologically, both TMPRSS2 and ACE2 are expressed in the lungs. TMPRSS2 is expressed mainly in sub-segmental bronchial branches and lung tissue, while ACE2 is mainly found in sub-segmental bronchial branches by transient secretory cell types [32,37]. Their colocalization is important and highlights the propensity of SARS-CoV-2 in this region. Colocalization of TMPRSS2 and ACE2 is also essential for effective infection of SARS-CoV-2, which enhances the efficiency of viral cellular entry due to proteolysis of the ACE2-protein S complex [37]. The importance of TMPRSS2 has been demonstrated in vivo studies in which.

TMPRSS2 deficiency in mice reduced viral particles in the lungs [38]. Also, it was reported that ADAM17 could also cleave ACE2, and it competed with TMPRSS2 [39]. Therefore, this evidence indicated the potential protective effect of ADAM17. However, with TMPRSS2, ADAM17 also regulates the ectodomain shedding of ACE2, which supports SARS-CoV-2 entry through endocytosis [40]. Therefore, further investigations are needed to delineate the exact role of ADAM17 in SARS-CoV-2 infection.

Overall, evidence shows the pivotal role of protein S priming by host proteases and inhibiting these proteases may hold a clue for COVID-19 therapy. Protein S cleavage allows viral fusion and entry to the cells, initiating infection and the viral reproduction process. These processes are also crucial in the pathogenesis of COVID-19-induced new-onset diabetes, which will be explained in the next section of this review.

## 3. ACE2: The link between SARS-CoV-2 and the key metabolic tissues

**Fig. 2:**
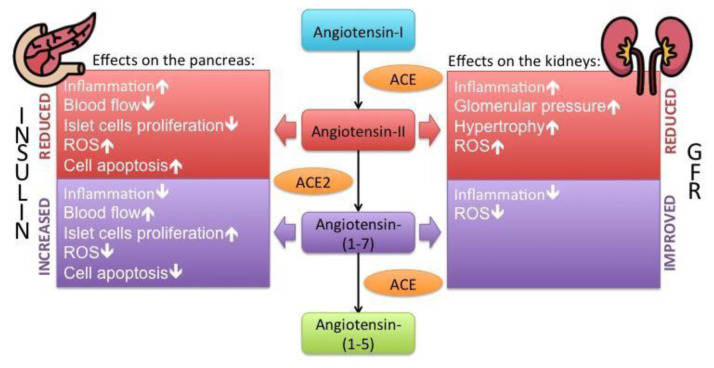
The role of ACE and ACE2 in the RAS signaling pathways. Both enzymes work in balance to maintain homeostasis in human body.

ACE2 is a unique, newly found enzyme that plays an important role as a compensatory enzyme in the pathogenic process of diabetes. Besides its well-known expression in the respiratory tract, ACE2 also presents in essential metabolic tissues such as the pancreas, liver, adipose tissue and kidney. ACE2 is localized in the acini and islets part of the pancreas, similar to ACE distribution [41]. A recent study suggested that ACE2 expression is slightly higher in the pancreas than in the lungs. Additionally, its expression occurs in both the exocrine and endocrine glands of the pancreas [42]. In the liver, ACE2 presents in hepatocytes, where it was found to be elevated in hepatic fibrosis and hypoxic condition of the liver, indicating its compensatory part for such fibrogenic diseases [43,44]. While in the kidney, ACE2 collocates with ACE on the apical surface of the proximal tubules and glomerulus [45]. Its presence in the vital metabolic tissues contributes to the normal regulation of the tissue renin-angiotensin system (RAS) signaling pathway and maintenance of the metabolism homeostasis (see Fig. 2, illustration adapted from Battle et al. [46]).

ACE2 inhibits angiotensin-II upregulation by degrading angiotensin-II into the primary products Ang-(1–7), which could counter the debilitating effects of RAS hyperactivity such as hyperglycemia, hypertension, cardiac dysfunction and fibrosis [47]. While angiotensin-II has vasoconstriction, pro-oxidant and inflammation effects, the products of ACE2 act on the Mas receptor to counteract such effects by inducing vasodilation, prostaglandin release and inhibition of norepinephrine secretion [48–50]. The vasodilatory effect is thought to result from the modulation of NO release by the Akt pathway by the angiotensin-(1–7). The NO modulation leads to compensatory impacts such as increased blood flow of the islet vessels during demanding functional conditions such as obesity, diabetes or merely high blood glucose peak [47].

Moreover, ACE2 also has an inhibitory effect on damaging islet factors, reactive oxygen species (ROS) and TGF-β. Previous studies imply that during the state of hyperglycemia, ROS has produced in pancreatic β-cells through the activation of NAD(P) H oxidase (NOX). The activation of NOX is induced by angiotensin-II and AT1 receptor interaction, eventually leading to pancreatic β-cells dysfunction [51]. Through its degrading angiotensin-II mechanism, ACE2 could prevent this damaging process and maintain the pancreatic β-cells morphology [52]. In line with that, ACE2 also preserves islet structure through the blockade of TGF-β by RAS inhibition. All these protective and compensatory mechanisms supposedly prevent islet fibrosis and function loss. Despite its lack of effect on basal insulin secretion, RAS inhibition by ACE2 could protect β-cells from damaging factors, thus improving insulin synthesis and secretion [47].

The evidence of the presence of ACE2 in essential metabolic tissues, especially the pancreas and its key role in SARS-CoV-2 infection shows a strong link that may lead to pancreatic injury hyperglycemia episodes or, even worse, new-onset diabetes. However, its pathogenesis of pancreatic damage remains controversial, and it is essential to uncover possible therapeutical intervention purposes. The established theory of ACE2 as a key entrance of SARS-CoV-2 indicates a more complicated possibility of the exact role of ACE2 in diabetes since it could act as a double-edged sword [47–53]. On the one hand, ACE2 expression is favorable for its protective mechanism in acute lung injury and compensatory effects in diabetes. However, on the other hand, its elevated expression may also facilitate more coronavirus entry into the host cells.

In response to that evidence, more researches are ongoing to elucidate a more explicit pathogenic process. Liu et al., in their cohort research, showed that 1–2% of nonsevere (without comorbidity and asymptomatic) and 17% of severe (with comorbidities and presenting symptoms) COVID-19 patients had a pancreatic injury [42]. Immunohistochemical staining in their study showed that both endocrine and exocrine glands of the pancreas expressed ACE2 quite significantly in COVID-19 patients. It was also suggested that patients in the study might have been experiencing pancreatic injury even before admission, indicating the coronavirus's rapid attack on the pancreatic cells. This acute damaging process leads to the acute onset of hyperglycemia, which becomes one possible reason for the higher risk of death in SARS-CoV-2 infection [42]. This result is also in line with Yang et al. report that SARS-CoV-infected patients presented with hyperglycemia, which might be resulted from the damaging process of the pancreatic islets through ACE2 [53]. The idea of COVID-19-induced new-onset diabetes is still very novel, and evidence-based theories are still limited. Nonetheless, further research has been undertaken better to understand the unique mechanisms and well-established therapeutical interventions.

## 4. SARS-CoV-2-induced new-onset diabetes

New-onset diabetes has been long established for more than a decade [54], and in recent days, it is coming to the surface again due to COVID-19. According to the term itself, new-onset means the symptoms occur for the first time in people without any history of diabetes. If left untreated, the symptoms manifest so acutely that they may develop into fatal complications such as ketoacidosis and hyperosmolarity [55,56]. Several conditions can induce this condition, including organ transplantation [57], the use of hypertensive drugs, thiazide diuretics and beta blockers [58], as well as a severe infection [53,55,56]. In the case of SARS-CoV-2 infection, however, diabetes and COVID-19 have a bidirectional relationship. Diabetes could exist previously as a comorbid and increase the risk of severe COVID-19, but it could also present for the first time as an acute onset. The latter case will require aggressive treatment, and its disease progression depends on the patient's clinical status [59].

Several studies illustrate a possible link between new-onset hyperglycemia and the severe coronavirus disease 2019 (COVID-19). Interestingly, this new-onset hyperglycemia is not associated with other risk factors, such as obesity, prediabetes, diabetes mellitus, or corticosteroid use [60]. Another finding by Li et al. states that COVID-19 patients who develop new-onset diabetes are known to have a higher mortality risk than COVID-19 patients who have had a history of diabetes or hyperglycemia [61]. Also, evidence regarding a high prevalence of diabetic ketoacidosis and hyperosmolarity has been documented in patients with COVID-19. Case reports suggest that COVID-19 can accelerate diabetic ketoacidosis (DKA) in subjects with new-onset hyperglycemia (diabetes) or pre-existing diabetes mellitus [62]. Early identification of DKA symptoms is needed to improve the prognosis of DKA related to COVID-19 [62]. Table 1 [63–65] summarizes the characteristics of new-onset diabetes in patients with COVID-19 that have been reported in several case reports.

However, the specific metabolic complications of COVID-19 are still not well defined. Therefore, an international diabetes research group initiated the CoviDIAB Project to conduct global records of diabetes patients related to Covid-19 (covidiab.edendrite. com). The purpose of recording this data is to define the phenotype of new-onset diabetes in patients with COVID-19. This condition is determined based on hyperglycemia, confirmed COVID-19, previous negative diabetes history, and a history of normal HbA1c levels. The registry will also be expanded to allow records of patients with preexisting diabetes who later present with severe acute metabolic disorders. So, this data is expected to discover the epidemiology and pathogenesis of diabetesrelated to COVID-19 and obtain instructions on the right treatment choice for patients [59].

### 4.1. Diabetic ketoacidosis as a possible complication of new-onset diabetes in COVID-19

Diabetic Ketoacidosis (DKA) is a complication that can cause morbidity and mortality in people with diabetes mellitus. DKA generally occurs due to decreasing insulin levels in the blood, which causes a decrease in glucose use and uncontrolled lipolysis, which in turn causes an excessive increase in ketone bodies and acidosis. This insulin deficiency condition occurs due to decreased secretion by pancreatic beta cells or increased insulin requirements triggered by infectious stressors and sepsis. The study results by Ahuja et al. stated that the strongest predisposing factor for acute DKA attacks was infection compared to other predisposing factors such as an inadequate insulin regimen, early presentation, or other unknown reasons [66].

As with many other diseases, COVID-19 could affect DKA patients by increasing the production of stress hormones and stimulating cytokines. Severe acute respiratory syndrome coronavirus-2 (SARS-CoV-2) utilizes binding to the angiotensin-converting enzyme 2 (ACE2) receptor on infected cells' membrane to enter the body's cells as a viral complex. ACE2 is found in many organs, such as the lungs, intestinal tissue, kidneys, heart and pancreas. ACE2 will convert angiotensin II to angiotensin I. The wide expression of ACE2 in these organs may explain the clinical symptoms of SARS. When talking about DKA that occurs in patients with COVID-19, it is known that ACE2 is expressed in the endocrine part of the pancreas. This evidence supports the statement that SARS-CoV-2 can enter the islet of the pancreas using ACE2 as its receptor. Therefore, a possible mechanism that plays a role in the development of DKA is the spread of ACE2 receptors by SARS-CoV-2 during this virus–host interaction, which can cause damage to pancreatic beta cells and subsequently interfere with their function. Furthermore, insulin deficiency can occur, leading to the development of acute diabetes [67,68].

In addition to direct beta cell damage, ACE2 expression on the surface of the pancreas is down regulated along with endocytosis of the ACE2-virus receptor complex. Reduced ACE2 expression can increase angiotensin II concentration, which cannot be converted to angiotensin I. In turn, the condition can inhibit insulin secretion [10,69]. The interaction between the virus that causes COVID-19 and the renin-angiotensin-aldosterone (RAAS) system may explain the pathophysiology that underlies DKA. These two factors are likely the basis of the acute deterioration of pancreatic beta cell function and the trigger for DKA in patients with COVID19. Studies on whether the nature of these changes is permanent or temporary are still to be carried out.

## 5. Possible therapeutic intervention

According to the latest studies, there are several recommendations regarding antidiabetic agents in COVID-19 patients. As discussed earlier, patients with COVID-19 can experience acute hyperglycemia. Clinicians must carry out glycemic control quickly, precisely and effectively to deal with this condition. Therefore, it is necessary to be careful in choosing the therapeutic modality based on its potential effectiveness and side effects. In their respective reviews, Lim et al. and Drucker have recommended glucagon-like peptide-1 receptor agonists (GLP-1Ras) for COVID-19 patients with mild to moderate symptoms because these agents can reduce glucose levels as well in outpatients [70,71]. However, the study results still do not support the recommendation to use this modality as a substitute for insulin in critically ill patients with type-2 diabetes mellitus and COVID-19, especially if therapy must be started in severe conditions.

GLP-1 receptor agonists (GLP1-RAs) or incretin-mimetics provide pharmacological levels of exogenous GLP1, which, analogous to the incretin hormone, have the effect of losing weight, inhibiting the release of glucagon, inhibiting appetite, and slowing gastric emptying [72,73]. GLP-1RAs have broad anti-inflammatory action when studied in animals with inflammation. This agent can also reduce systemic inflammatory biomarkers in human subjects with type 2 diabetes mellitus and obesity [74]. Several studies have shown that GLP-1RAs can reduce lung inflammation, decrease cytokine production and maintain lung function in mice with experimental lung injury [75–77]. GLP-1RAs have been shown to reduce pulmonary type 2 immune cytokine responses and lung damage levels in mice with respiratory syncytial virus (RSV) infection isolated from a hospitalized infant with severe lower respiratory tract infection and bronchiolitis [78]. Liraglutide, a GLP-1Ras, has a good safety and effectiveness profile when used as acute control of perioperative blood glucose in adult subjects undergoing elective cardiovascular surgery [79]. Also, liraglutide has been reported to improve cardiovascular outcomes in diabetic patients. It has a minimal risk of causing hypoglycemia, so it would be great if the administration of this agent could be further investigated in COVID-19 patients with diabetes [80].

Furthermore, based on Lim et al. [70] and Drucker's [71] recommendation, the use of insulin can be suggested. Insulin has become the glycemic control agent of choice for hospitalized COVID-19 patients, and its use is mandatory for critically ill patients. In its guideline, the American Diabetes Association (ADA) states that basal insulin or basal-corrected insulin regimen plus a bolus is a therapeutic option for hospitalized patients who are not seriously ill. Meanwhile, continuous intravenous insulin infusion is becoming a more recommended treatment for critically ill patients in the ICU. The expected target blood glucose for critically ill and non-critically ill patients ranges from 140 mg/dL to 180 mg/dL (7.8–10.0 mmol/L) [81].

Regardless of the ADA recommendations, although insulin treatment is the choice for diabetic patients with severe COVID-19 [81], a study in Wuhan, China, reported a worse prognosis based on clinical and laboratory data in patients using insulin than with patients using metformin [82]. However, these results should still be examined with caution because of the possible confounding, as insulin treatment is generally used in more severe diabetes patients. Other research supports this hypothesis that insulin infusion is an effective method to achieve the expected glycemic control and could reduce the severity and mortality in diabetic patients with COVID-19 [83].

Several studies have also shown that insulin administration can reduce urine ACE2, kidney ADAM-17 and kidney ACE2 in type-1 diabetes mouse models [84,85]. Insulin is known to act as an immunomodulatory agent and an additional anti-inflammatory agent. These roles include blocking the NF Kβ signaling pathway, reducing TNF-α levels and disrupting neutrophil chemotaxis [86].

Palermo et al. reviewed the recommendations for DKA treatment in COVID-19 patients [87]. The subcutaneous insulin regimen is the primary modality emphasized in the article. Blood glucose and ketone bodies in COVID-19 patients with hyperglycemia should be monitored regularly [88]. There are no specific guidelines regarding fluid and electrolyte management in patients with COVID-19 and diabetes mellitus. However, several articles can be referred to for management considerations [89,90].

Preclinical studies report the anti-inflammatory role of metformin, wherein metformin can reduce inflammatory biomarkers' levels in the circulation of patients with type-1 diabetes mellitus [91]. In a Chinese study comparing hospital mortality among COVID-19 patients with diabetes, the hospital mortality rate was significantly higher in patients who did not receive metformin than in those who received metformin (12.3% vs.2.9%; P = 0.01) [92]. However, these findings may have a selection bias because patients with severe respiratory problems cannot receive metformin. When discussed from a molecular perspective, 50-AMP-activated protein kinase (AMPK) is the main effector of metformin's pharmacological action. This molecule appears to have a role in regulating the stability and expression of ACE2. Metformin can increase the expression of ACE2 and phosphorylation to the Ser680 residue in HUVEC cells. In addition, through AMPK, metformin also mediates ACE2 phosphorylation, thereby increasing the stability of ACE2. This process occurs through the inhibition of ubiquitination and degradation of its proteasomes. Therefore, theoretically, metformin might increase the amount of ACE2 in the respiratory tract, thereby increasing the chance for SARS-CoV2 to enter cells [93–95]. The clinical evidence to prove this theory requires further investigation.

Nonetheless, it cannot be denied that metformin does have protective effects due to its multiple molecular mechanisms in the vascular. Metformin could halt the activation of platelet and the release of mitochondrial DNA and suppress interaction between leukocytes and endothelium, thus reducing endothelial inflammation. These mechanisms prevent vein and artery thrombosis, conferring vascular protection [96]. With its broad protective mechanism, metformin is considered one of the primary choices of infusion medication with micro needles in the newest technology of diabetes treatment [97].

## 6. Conclusion

Diabetes mellitus and COVID-19 are the challenging diseases faced in recent days. New-onset diabetes is one problem induced by SARS-CoV-2 infection in nondiabetic patients. ACE2 is the critical key factor that possibly plays a vital role in new-onset diabetes, as DM and COVID-19 are interrelated with ACE2 in molecular pathogenesis. Treatments in acute hyperglycemic conditions are still controversial since there are still discrepancies regarding the results in the field. A careful decision based on the patient's current clinical condition and comorbidities is needed to make rational choices of treatment, which could then provide a precise and good outcome.

## Figures and Tables

**Table 1:** Characteristics of new-onset diabetes in COVID-19 patients from several case reports.

Reference	Gender	Age	BMI	Patient's History
Chee et al. [63]	Male	37 y.o	22.6 -kg/m^2^	A previously healthy man with no evidence of Insulin resistance
			–	The patient has symptoms of fever, vomiting, polyuria and polydipsia one week before admission to the hospital
			–	Abnormality in the physical examination: mildly tachycardic.
			–	Laboratory results: high blood glucose, high anion gap metabolic acidosis and ketonemia confirmed the patient to be in DKA.
Haidil et al. [64]	Male	47 y.o	26.3 -kg/m^2^	The patient was initially not known to have diabetes but had nocturia, fatigue, and general body aches four days before admission.
			–	Abnormalities in the physical examination: Tachycardic and Tachipneic
			–	Laboratory results: hyperglycemia, high anion gap metabolic acidosis and ketonuria, confirming the diagnosis of DKA
Heaney et al. [65]	Male	54 y.o	42.56 -kg/m^2^	The patient experienced fatigue for three weeks, which later developed into shortness of breath and coughing one week before being admitted to the hospital.
			–	The patient has a history of kidney stones, hypertension, testicular hypofunction and erectile dysfunction
			–	Abnormalities in the physical examination: ill, tachypneic, tachycardic.
			–	Laboratory results: high blood glucose, anion gap metabolic acidosis, and ketonuria confirming the diagnosis of DKA

## References

[1] World Health Organization Global report on diabetes Switzerland WHO Press 2016 25 33

[2] Shi Y Wang G Cai X Deng J Zheng L Zhu H An overview of COVID-19 J Zhejiang Univ - Sci B 2020 21 5 343 60 32425000 10.1631/jzus.B2000083 PMC7205601

[3] World Health Organization COVID-19 weekly epidemiological update Published January, 3, 2021. Updated January 3, 2021 Accessed January 7, 2021 https://apps.who.int/iris/handle/10665/339547

[4] Baj J Karakuła-Juchnowicz H Teresiński G Buszewicz G Ciesielka M Sitarz E COVID-19: specific and non-specific clinical manifestations and symptoms: the current state of knowledge J Clin Med 2020 9 6 1753 32516940 10.3390/jcm9061753 PMC7356953

[5] Critchley JA Carey IM Harris T DeWilde S Hosking FJ Cook DG Glycemic control and risk of infections among people with type 1 or type 2 diabetes in a large primary care cohort study Diabetes Care 2018 41 10 2127 35 30104296 10.2337/dc18-0287

[6] Carey IM Critchley JA DeWilde S Harris T Hosking FJ Cook DG Risk of infection in type 1 and type 2 diabetes compared with the general population: a matched cohort study Diabetes Care 2018 41 3 513 21 29330152 10.2337/dc17-2131

[7] Yang X Yu Y Xu J Shu H Liu H Wu Y Clinical course and outcomes of critically ill patients with SARS-CoV-2 pneumonia in Wuhan, China: a single-centered, retrospective, observational study Lancet Respir Med 2020 8 5 475 81 32105632 10.1016/S2213-2600(20)30079-5 PMC7102538

[8] Zhang J Dong X Cao Y Yuan Y Yang Y Yan Y Clinical characteristics of 140 patients infected with SARS-CoV-2 in Wuhan, China Allergy 2020 75 7 1730 41 32077115 10.1111/all.14238

[9] Richardson S Hirsch JS Narasimhan M Crawford JM McGinn T Davidson KW Presenting characteristics, comorbidities, and outcomes among 5700 patients hospitalized with COVID-19 in the New York City area JAMA 2020 323 20 2052 9 32320003 10.1001/jama.2020.6775 PMC7177629

[10] Guo W Li M Dong Y Zhou H Zhang Z Tian C Diabetes is a risk factor for the progression and prognosis of COVID-19 Diabetes Metab Res Rev 2020 36 7 e3319 32233013 10.1002/dmrr.3319 PMC7228407

[11] Alsadhan I Alruwashid S Alhamad M Alajmi S Alshehri S Alfadhli E Diabetic ketoacidosis precipitated by Coronavirus disease 2019 infection: case series Curr Ther Res 2020 93 100609 33132404 10.1016/j.curtheres.2020.100609 PMC7590633

[12] Suwanwongse K Shabarek N Newly diagnosed diabetes mellitus, DKA, and COVID-19: causality or coincidence? A report of three cases J Med Virol 2021 93 2 1150 3 32706395 10.1002/jmv.26339 PMC7404645

[13] Tipnis SR Hooper NM Hyde R Karran E Christie G Turner AJ A human homolog of angiotensin-converting enzyme. Cloning and functional expression as a captopril-insensitive carboxypeptidase J Biol Chem 2000 275 33238 43 10924499 10.1074/jbc.M002615200

[14] Wan Y Shang J Graham R Baric RS Li F Receptor recognition by the novel coronavirus from Wuhan: an analysis based on decade-long structural studies of SARS coronavirus J Virol 2020 94 7 001277 e220 10.1128/JVI.00127-20 PMC7081895 31996437

[15] Hoffmann M Kleine-Weber H Schroeder S Krüger N Herrler T Erichsen S SARS-CoV-2 cell entry depends on ACE2 and TMPRSS2 and is blocked by a clinically proven protease inhibitor Cell 2020 181 2 271 80 32142651 10.1016/j.cell.2020.02.052 PMC7102627

[16] Li Y Zhou W Yang L You R Physiological and pathological regulation of ACE2, the SARS-CoV-2 receptor Pharmacol Res 2020 157 104833 32302706 10.1016/j.phrs.2020.104833 PMC7194807

[17] Zhou F Yu T Du R Fan G Liu Y Liu Z Clinical course and risk factors for mortality of adult inpatients with COVID-19 in Wuhan, China: a retrospective cohort study Lancet (London, England) 2020 395 10229 1054 62 32171076 10.1016/S0140-6736(20)30566-3 PMC7270627

[18] Scialo F Daniele A Amato F Pastore L Matera MG Cazzola M ACE2: the major cell entry receptor for SARS-CoV-2 Lung 2020 198 6 867 77 33170317 10.1007/s00408-020-00408-4 PMC7653219

[19] Kuhn JH Li W Choe H Farzan M Angiotensin-converting enzyme 2: a functional receptor for SARS coronavirus Cell Mol Life Sci 2004 61 21 2738 43 15549175 10.1007/s00018-004-4242-5 PMC7079798

[20] Li W Moore MJ Vasilieva N Sui J Wong SK Berne MA Angiotensin-converting enzyme 2 is a functional receptor for the SARS coronavirus Nature 2003 426 6965 450 4 14647384 10.1038/nature02145 PMC7095016

[21] Ng ML Tan SH See EE Ooi EE Ling AE Proliferative growth of SARS coronavirus in Vero E6 cells J Gen Virol 2003 84 12 3291 303 14645910 10.1099/vir.0.19505-0

[22] de Wilde AH Raj VS Oudshoorn D Bestebroer TM van Nieuwkoop S Limpens RWAL MERS-coronavirus replication induces severe in vitro cytopathology and is strongly inhibited by cyclosporin A or interferon-α treatment J Gen Virol 2013 94 8 1749 60 23620378 10.1099/vir.0.052910-0 PMC3749523

[23] Tseng CTK Huang C Newman P Wang N Narayanan K Watts DM Severe acute respiratory syndrome coronavirus infection of mice transgenic for the human Angiotensinconverting enzyme 2 virus receptor J Virol 2007 81 3 1162 73 17108019 10.1128/JVI.01702-06 PMC1797529

[24] McCray PB Jr Pewe L Wohlford-Lenane C Hickey M Manzel L Shi L Lethal infection of K18-hACE2 mice infected with severe acute respiratory syndrome coronavirus J Virol 2007 81 2 813 21 17079315 10.1128/JVI.02012-06 PMC1797474

[25] Yang XH Deng W Tong Z Liu YX Zhang LF Zhu H Mice transgenic for human angiotensin-converting enzyme 2 provide a model for SARS coronavirus infection Comp Med 2007 57 5 450 9 17974127

[26] Pang XC Zhang HX Zhang Z Rinkiko S Cui YM Zhu YZ The two-Way switch role of ACE2 in the treatment of novel coronavirus pneumonia and underlying comorbidities Molecules 2020 26 1 142 33396184 10.3390/molecules26010142 PMC7794970

[27] Kuba K Imai Y Rao S Gao H Guo F Guan B A crucial role of angiotensin converting enzyme 2 (ACE2) in SARS coronavirus-induced lung injury Nat Med [Internet] 2005 11 8 875 9 16007097 10.1038/nm1267 PMC7095783

[28] Zhang H Penninger JM Li Y Zhong N Slutsky AS Angiotensin-converting enzyme 2 (ACE2) as a SARS-CoV-2 receptor: molecular mechanisms and potential therapeutic target Intensive Care Med 2020 46 4 586 90 32125455 10.1007/s00134-020-05985-9 PMC7079879

[29] Li G De Clercq E Therapeutic options for the 2019 novel coronavirus (2019-nCoV) Nature reviews Drug discovery England 2020 19 149 50 10.1038/d41573-020-00016-0 32127666

[30] Glowacka I Bertram S Müller MA Allen P Soilleux E Pfefferle S Evidence that TMPRSS2 activates the severe acute respiratory syndrome coronavirus spike protein for membrane fusion and reduces viral control by the humoral immune response J Virol 2011 85 9 4122 34 21325420 10.1128/JVI.02232-10 PMC3126222

[31] Matsuyama S Nagata N Shirato K Kawase M Takeda M Taguchi F Efficient activation of the severe acute respiratory syndrome coronavirus spike protein by the transmembrane protease TMPRSS2 J Virol 2010 84 24 12658 64 20926566 10.1128/JVI.01542-10 PMC3004351

[32] Shulla A Heald-Sargent T Subramanya G Zhao J Perlman S Gallagher T A transmembrane serine protease is linked to the severe acute respiratory syndrome coronavirus receptor and activates virus entry J Virol 2011 85 2 873 82 21068237 10.1128/JVI.02062-10 PMC3020023

[33] Simmons G Gosalia DN Rennekamp AJ Reeves JD Diamond SL Bates P Inhibitors of cathepsin L prevent severe acute respiratory syndrome coronavirus entry Proc Natl Acad Sci U S A 2005 102 33 11876 81 16081529 10.1073/pnas.0505577102 PMC1188015

[34] Lu G Wang Q Gao GF Bat-to-human: spike features determining host jump of coronaviruses SARS-CoV, MERS-CoV, and beyond Trends Microbiol 2015 23 8 468 78 26206723 10.1016/j.tim.2015.06.003 PMC7125587

[35] Izaguirre G The proteolytic regulation of virus cell entry by Furin and other proprotein convertases Viruses 2019 11 9 837 31505793 10.3390/v11090837 PMC6784293

[36] Zhou P Yang XL Wang XG Hu B Zhang L Zhang W A pneumonia outbreak associated with a new coronavirus of probable bat origin Nature 2020 579 7798 270 3 32015507 10.1038/s41586-020-2012-7 PMC7095418

[37] Lukassen S Chua RL Trefzer T Kahn NC Schneider MA Muley T SARS-CoV-2 receptor ACE2 and TMPRSS2 are primarily expressed in bronchial transient secretory cells EMBO J 2020 39 10 e105114 32246845 10.15252/embj.20105114 PMC7232010

[38] Iwata-Yoshikawa N Okamura T Shimizu Y Hasegawa H Takeda M Nagata N TMPRSS2 contributes to virus spread and immunopathology in the airways of Murine Models after Coronavirus infection J Virol 2019 93 6 e01815 8 30626688 10.1128/JVI.01815-18 PMC6401451

[39] Heurich A Hofmann-Winkler H Gierer S Liepold T Jahn O Pöhlmann S TMPRSS2 and ADAM17 cleave ACE2 differentially and only proteolysis by TMPRSS2 augments entry driven by the severe acute respiratory syndrome coronavirus spike protein J Virol 2014 88 2 1293 307 24227843 10.1128/JVI.02202-13 PMC3911672

[40] Xiao L Sakagami H Miwa N ACE2: the key molecule for understanding the pathophysiology of severe and critical conditions of COVID-19: demon or angel? Viruses 2020 12 5 491 32354022 10.3390/v12050491 PMC7290508

[41] Ye M Wysocki J William J Soler MJ Cokic I Batlle D Glomerular localization and expression of angiotensin-converting enzyme 2 and angiotensin-converting enzyme: implications for albuminuria in diabetes J Am Soc Nephrol 2006 17 3067 75 17021266 10.1681/ASN.2006050423

[42] Liu F Long X Zhang B Zhang W Chen X Zhang Z ACE2 expression in pancreas may cause pancreatic damage after SARS-CoV-2 infection Clin Gastroenterol Hepatol 2020 18 9 2128 30 32334082 10.1016/j.cgh.2020.04.040 PMC7194639

[43] Paizis G Tikellis C Cooper ME Schembri JM Lew RA Smith AI Chronic liver injury in rats and humans upregulates the novel enzyme angiotensin converting enzyme 2 Gut 2005 54 1790 6 16166274 10.1136/gut.2004.062398 PMC1774784

[44] Herath C Warner FJ Lubel J Dean RJ Jia Z Lew RA Upregulation of hepatic angiotensin-converting enzyme 2 (ACE2) and angiotensin-(1–7) levels in experimental billiary fibrosis J Hepatol 2007 47 387 95 17532087 10.1016/j.jhep.2007.03.008 PMC7114685

[45] Tikellis C Wookey PJ Candido R Andrikopoulos S Thomas MC Cooper ME Improved islet morphology after blockade of the renin-angiotensin system in the ZDF rat Diabetes 2004 53 989 97 15047614 10.2337/diabetes.53.4.989

[46] Batlle D Jose Soler M Ye M ACE2 and diabetes: ACE of ACEs? Diabetes 2010 59 12 2994 6 21115782 10.2337/db10-1205 PMC2992757

[47] Bindom SM Lazartigues E The sweeter side of ACE2: physiological evidence for a role in diabetes Mol Cell Endocrinol 2009 302 2 193 202 18948167 10.1016/j.mce.2008.09.020 PMC2676688

[48] Almeida AP Frábregas BC Madureira MM Santos RJS Campagnole-Santos MJ Santos RAS Angiotensin-(1–7) potentiates the coronary vasodilatatory effect of bradykinin in the isolated rat heart Braz J Med Biol Res 2000 33 709 13 10829099 10.1590/s0100-879x2000000600012

[49] Gironacci MM Valera MS Yujnovsky I Pena C Angiotensin-(1–7) inhibitory mechanism of norepinephrine release in hypertensive rats Hypertension 2004 44 783 7 15381673 10.1161/01.HYP.0000143850.73831.9d

[50] Sampaio WO Souza dos Santos RA Faria-Silva R da Mata Machado LT Schiffrin EL Touyz RM Angiotensin-(1–7) through receptor Mas mediates endothelial nitric oxide synthase activation via Akt-dependent pathways Hypertension 2007 49 185 92 17116756 10.1161/01.HYP.0000251865.35728.2f

[51] Nakayama M Inoguchi T Sonta T Maeda Y Sasaki S Sawada F Increased expression of NAD(P)H oxidase in islets of animal models of type 2 diabetes and its improvement by an AT1 receptor antagonist Biochem Biophys Res Commun 2005 332 927 33 15922295 10.1016/j.bbrc.2005.05.065

[52] Grobe JL Der SS Stewart JM Meszaros JG Raizada MK Katovich MJ ACE2 overexpression inhibits hypoxia-induced collagen production by cardiac fibroblasts Clin Sci 2007 113 357 64 10.1042/CS20070160 17600530

[53] Yang JK Lin SS Ji XJ Guo LM Binding of SARS coronavirus to its receptor damages islets and causes acute diabetes Acta Diabetol 2010 47 3 193 9 19333547 10.1007/s00592-009-0109-4 PMC7088164

[54] Smith NL Barzilay JI Kronmal R Lumley T Enquobahrie D Psaty BM New-onset diabetes and risk of all-cause and cardiovascular mortality: the Cardiovascular Health Study Diabetes Care 2006 Sep 29 9 2012 7 16936145 10.2337/dc06-0574

[55] Winn SP Oo ZT Htun NN Soe MHP Aung MM Diabetic ketoacidosis in coronavirus disease patients with type 2 diabetes mellitus Cureus 2020 12 8 e9731 32953287 10.7759/cureus.9731 PMC7491247

[56] Palermo NE Sadhu AR McDonnell ME Diabetic ketoacidosis in COVID-19: unique concerns and considerations J Clin Endocrinol Metab 2020 105 8 1 11 32556147 10.1210/clinem/dgaa360 PMC7337869

[57] Pham PT Pham PM Pham SV Pham PA Pham PC New onset diabetes after transplantation (NODAT): an overview Diabetes, Metab Syndrome Obes Targets Ther 2011 4 175 10.2147/DMSO.S19027 PMC3131798 21760734

[58] Mancia G Grassi G Zanchetti A New-onset diabetes and antihypertensive drugs J Hypertens 2006 24 1 3 10 16331092 10.1097/01.hjh.0000194119.42722.21

[59] Rubino F Amiel SA Zimmet P New-onset diabetes in covid-19 N Engl J Med 2020 383 8 789 90 32530585 10.1056/NEJMc2018688 PMC7304415

[60] Singh AK Singh R Hyperglycemia without diabetes and new-onset diabetes are both associated with poorer outcomes in COVID-19 Diabetes Res Clin Pract 2020 167 108382 32853686 10.1016/j.diabres.2020.108382 PMC7445123

[61] Li H Tian S Chen T Cui Z Shi N Zhong X Newly diagnosed diabetes is associated with a higher risk of mortality than known diabetes in hospitalized patients with COVID-19 Diabetes Obes Metabol 2020 22 10 1897 906 10.1111/dom.14099 PMC7283710 32469464

[62] Reddy PK Kuchay MS Mehta Y Mishra SK Diabetic ketoacidosis precipitated by COVID-19: a report of two cases and review of literature Diabetes Metabol Syndr 2020 14 1459 62 10.1016/j.dsx.2020.07.050 PMC7395228 32771918

[63] Chee YJ Ng SJH Yeoh E Diabetic ketoacidosis precipitated by Covid-19 in a patient with newly diagnosed diabetes mellitus Diabetes Res Clin Pract 2020 164 108166 32339533 10.1016/j.diabres.2020.108166 PMC7194589

[64] Hadil AAO Eman S Bashayer Zuhair AS Anwar J Diabetic ketoacidosis and new onset diabetes mellitus precipitated by COVID-19 infection JOJ Case Stud 2020 11 3 555815

[65] Heaney AI Griffin GD Simon EL Newly diagnosed diabetes and diabetic ketoacidosis precipitated by COVID-19 infection Am J Emerg Med 2020 38 11 2491e3 2491.e4 10.1016/j.ajem.2020.05.114 PMC7274947 32536476

[66] Ahuja W Kumar N Kumar S Precipitating risk factors, clinical presentation, and outcome of diabetic ketoacidosis in patients with type 1 diabetes Cureus 2019 11 5 e4789 31372327 10.7759/cureus.4789 PMC6669022

[67] Bornstein SR Dalan R Hopkins D Endocrine and metabolic link to coronavirus infection Nat Rev Endocrinol 2020 16 297 8 32242089 10.1038/s41574-020-0353-9 PMC7113912

[68] Unsworth R Wallace S Oliver NS Yeung S Kshirsagar A Naidu H New-onset type 1 diabetes in children during COVID-19: multicenter regional findings in the UK Diabetes Care 2020 43 11 e170 1 32816997 10.2337/dc20-1551

[69] Roca-Ho H Riera M Palau V Pascual J Soler MJ Characterization of ACE and ACE2 expression within different organs of the NOD mouse Int J Mol Sci 2017 18 3 563 28273875 10.3390/ijms18030563 PMC5372579

[70] Lim S Bae JH Kwon HS COVID-19 and diabetes mellitus: from pathophysiology to clinical management Nat Rev Endocrinol 2021 17 11 30 33188364 10.1038/s41574-020-00435-4 PMC7664589

[71] Drucker DJ Coronavirus infections and type 2 diabetes–shared pathways with therapeutic implications Endocr Rev 2020 41 457 70 10.1210/endrev/bnaa011 PMC7184382 32294179

[72] Chiefari E Capula C Vero A Oliverio R Puccio L Liguori R Add-on treatment with liraglutide improves glycemic control in patients with type 2 diabetes on Metformin therapy Diabetes Technol Therapeut 2015 17 468 74 10.1089/dia.2014.0412 25844858

[73] Mirabelli M Chiefari E Caroleo P Arcidiacono B Corigliano DM Giuliano S Longterm effectiveness of Liraglutide for weight management and glycemic control in type 2 diabetes Int J Environ Res Publ Health 2019 17 207 10.3390/ijerph17010207 PMC6981922 31892206

[74] Drucker DJ Mechanisms of action and therapeutic application of glucagon-like peptide-1 Cell Metabol 2018 27 740 56 10.1016/j.cmet.2018.03.001 29617641

[75] Viby NE Isidor MS Buggeskov KB Poulsen SS Hansen JB Kissow H Glucagon-like peptide-1 (GLP-1) reduces mortality and improves lung function in a model of experimental obstructive lung disease in female mice Endocrinology 2013 154 4503 11 24092637 10.1210/en.2013-1666

[76] Toki S Goleniewska K Reiss S Zhang J Bloodworth MH Stier MT Glucagon-like peptide 1 signaling inhibits allergen-induced lung IL-33 release and reduces group 2 innate lymphoid cell cytokine production in vivo J Allergy Clin Immunol 2018 142 1515 1528e1518 29331643 10.1016/j.jaci.2017.11.043 PMC9639650

[77] Zhou F Zhang Y Chen J Hu X Xu Y Liraglutide attenuates lipopolysaccharide-induced acute lung injury in mice Eur J Pharmacol 2016 791 735 40 27756605 10.1016/j.ejphar.2016.10.016

[78] Bloodworth MH Rusznak M Pfister CC Zhang J Bastarache L Calvillo SA Glucagon-like peptide 1 receptor signaling attenuates respiratory syncytial virus-induced type 2 responses and immunopathology J Allergy Clin Immunol 2018 142 683 687e612 29678751 10.1016/j.jaci.2018.01.053 PMC6078807

[79] Hulst AH Visscher MJ Godfried MB Thiel B Gerritse BM Scohy TV Liraglutide for perioperative management of hyperglycaemia in cardiac surgery patients: a multicentre randomized superiority trial Diabetes Obes Metabol 2020 22 557 65 10.1111/dom.13927 PMC7079116 31749275

[80] Marso SP Daniels GH Brown-Frandsen K Kristensen P Mann JFE Nauck MA Liraglutide and cardiovascular outcomes in type 2 diabetes N Engl J Med 2016 375 311 22 27295427 10.1056/NEJMoa1603827 PMC4985288

[81] American Diabetes Association Diabetes care in the hospital: standards of medical care in diabetes-2020 Diabetes Care 2020 43 Suppl 1 S193 202 31862758 10.2337/dc20-S015

[82] Chen Y Yang D Cheng B Chen J Peng A Yang C Clinical characteristics and outcomes of patients with diabetes and COVID-19 in association with glucose-lowering medication Diabetes Care 2020 43 1399 407 32409498 10.2337/dc20-0660

[83] Sardu C D'Onofrio N Balestrieri ML Barbieri M Rizzo MR Messina V Outcomes in patients with hyperglycemia affected by COVID-19: can we do more on glycemic control? Diabetes Care 2020 43 7 1408 15 32430456 10.2337/dc20-0723 PMC7305003

[84] Riera M Marquez E Clotet S Gimeno J Roca-Ho H Lloreta J Effect of insulin on ACE2 activity and kidney function in the non-obese diabetic mouse PLoS One 2014 9 e84683 24400109 10.1371/journal.pone.0084683 PMC3882249

[85] Salem ESB Grobe N Elased KM Insulin treatment attenuates renal ADAM17 and ACE2 shedding in diabetic Akita mice Am J Physiol Ren Physiol 2014 306 F629 39 10.1152/ajprenal.00516.2013 PMC3949038 24452639

[86] Honiden S Gong MN Diabetes, insulin, and development of acute lung injury Crit Care Med 2009 37 2455 64 19531947 10.1097/CCM.0b013e3181a0fea5 PMC3103784

[87] Palermo NE Sadhu AR McDonnell ME Diabetic ketoacidosis in COVID-19: unique concerns and considerations J Clin Endocrinol Metab 2020 105 2819 29 10.1210/clinem/dgaa360 PMC7337869 32556147

[88] Khunti K Del Prato S Mathieu C Kahn SE Gabbay RA Buse JB COVID-19, hyperglycemia, and new-onset diabetes Diabetes Care 2021 44 12 2645 55 34625431 10.2337/dc21-1318 PMC8669536

[89] Hasanin A Mostafa M Evaluation of fluid responsiveness during COVID-19 pandemic: what are the remaining choices? J Anesth 2020 34 758 64 32451626 10.1007/s00540-020-02801-y PMC7246295

[90] Khan AA Ata F Munir W Yousaf Z Fluid replacement versus fluid restriction in COVID19 associated hyponatremia Cureus 2020 12 e9059 32782878 10.7759/cureus.9059 PMC7413320

[91] Cameron AR Anti-inflammatory effects of metformin irrespective of diabetes status Circ Res 2016 119 652 65 27418629 10.1161/CIRCRESAHA.116.308445 PMC4990459

[92] Luo P Metformin treatment was associated with decreased mortality in COVID-19 patients with diabetes in a retrospective analysis Am J Trop Med Hyg 2020 103 69 72 32446312 10.4269/ajtmh.20-0375 PMC7356425

[93] Zhang J Dong J Martin M He M Gongol B Marin TL AMP-activated protein kinase phosphorylation of angiotensin-converting enzyme 2 in endothelium mitigates pulmonary hypertension Am J Respir Crit Care Med 2018 198 4 509 20 29570986 10.1164/rccm.201712-2570OC PMC6118028

[94] Ursini F Ciaffi J Landini MP Meliconi R COVID-19 and diabetes: is metformin a friend or foe? Diabetes Res Clin Pract 2020 164 108167 32339534 10.1016/j.diabres.2020.108167 PMC7195096

[95] Varghese E Samuel SM Liskova A Kubatka P Büsselberg D Diabetes and coronavirus (SARS-CoV-2): molecular mechanism of Metformin intervention and the scientific basis of drug repurposing PLoS Pathog 2021 17 6 e1009634 34157054 10.1371/journal.ppat.1009634 PMC8219155

[96] Samuel SM Varghese E Büsselberg D Therapeutic potential of metformin in COVID-19: Reasoning for its protective role Trends Microbiol 2021 29 10 894 907 33785249 10.1016/j.tim.2021.03.004 PMC7955932

[97] Metwally AA Mehta P Johnson BS Nagarjuna A Snyder MP Covid-19–induced newonset diabetes: trends and technologies Diabetes 2021 70 12 2733 44 34686519 10.2337/dbi21-0029 PMC8660988

